# Network-Based Elucidation of Human Disease Similarities Reveals Common Functional Modules Enriched for Pluripotent Drug Targets

**DOI:** 10.1371/journal.pcbi.1000662

**Published:** 2010-02-05

**Authors:** Silpa Suthram, Joel T. Dudley, Annie P. Chiang, Rong Chen, Trevor J. Hastie, Atul J. Butte

**Affiliations:** 1 Stanford Center for Biomedical Informatics Research, Stanford University, Stanford, California, United States of America; 2 Department of Pediatrics, Stanford University, Stanford, California, United States of America; 3 Lucile Packard Children's Hospital, Palo Alto, California, United States of America; 4 Department of Statistics, Stanford University, Stanford, California, United States of America; Bar Ilan University, Israel

## Abstract

Current work in elucidating relationships between diseases has largely been based on pre-existing knowledge of disease genes. Consequently, these studies are limited in their discovery of new and unknown disease relationships. We present the first quantitative framework to compare and contrast diseases by an integrated analysis of disease-related mRNA expression data and the human protein interaction network. We identified 4,620 functional modules in the human protein network and provided a quantitative metric to record their responses in 54 diseases leading to 138 significant similarities between diseases. Fourteen of the significant disease correlations also shared common drugs, supporting the hypothesis that similar diseases can be treated by the same drugs, allowing us to make predictions for new uses of existing drugs. Finally, we also identified 59 modules that were dysregulated in at least half of the diseases, representing a common disease-state “signature”. These modules were significantly enriched for genes that are known to be drug targets. Interestingly, drugs known to target these genes/proteins are already known to treat significantly more diseases than drugs targeting other genes/proteins, highlighting the importance of these core modules as prime therapeutic opportunities.

## Author Summary

Many human diseases are related to each other through shared causes or even shared pathology. Knowledge of these relationships has long been exploited to treat similar diseases with the same therapies. However, most of the traditional approaches to discover these relationships have depended on subjective measures, such as similarity in symptoms, or incomplete knowledge, such as genes with mutations. Here we present the first approach integrating high-throughput datasets such as mRNA expression and large-scale protein-protein interaction networks to discover human disease relationships in a systematic and quantitative way. We discover 138 significant pathological similarities between 54 human diseases ranging from lung cancer, schizophrenia, and malaria. We also discovered a set of common pathways and processes within the cell that are dysregulated in at least half of the diseases. We infer that these processes correspond to a common response of the human system to a disease state. Interestingly, we find that many of the proteins in these pathways are already known to be targets of existing drugs. In fact, the drugs corresponding to these proteins are known to treat significantly more diseases than expected by chance highlighting the importance of these common molecular pathological pathways as prime therapeutic opportunities.

## Introduction

Our understanding of the human disease state is incomplete without the knowledge of how various diseases relate to each other. Relationships between diseases have been used to gain insights into the etiology and pathogenesis of similar diseases [1]. Study of disease similarities has also led to the discovery of new causal genes for diseases [2],[3]. Moreover, similarities between biological concepts such as genes have been used successfully in gene function prediction [4]. However, most of the early work on finding disease-similarity has been limited to studying the clinical phenotypes of the diseases. For instance, similarities in disease symptoms and pathological results have been used to ascertain similarities between Alzheimer's disease and vascular dementia [1]. These methods are not quantitative and cannot be used to compare the relative similarities between diseases. More recently, scientists have been able to explore the genetic similarity between diseases because of the availability of large-scale knowledge-bases such as the Online Mendelian Inheritance in Man (OMIM) [5]. In 2007, Goh and colleagues created the first “Diseasome”, a network of human diseases [6]. This network consisted of human diseases/disorders as nodes and two diseases were joined by a link if they shared known disease genes (data obtained from OMIM). Van Driel *et al.* [7] inferred disease-disease associations by an automated text mining of OMIM descriptions. Liu *et al.* [8] mined for disease etiologies from the Medical Subject Headings (MESH) [9] vocabulary and used it to reveal similarities between diseases. Although the above studies provided comprehensive views of disease interrelationships, they were mainly studying monogenic disorders and generally ignored the effect of the environment on these and other, more complex, diseases. They also relied heavily on information that is already known, such as known disease genes or known pathways. As a result, they were limited in their ability to uncover hitherto unknown relations between diseases.

Advances in high-throughput molecular assay technologies, accompanied by declining per-sample costs, have given rise to numerous public repositories of biomolecular data such as mRNA expression profiles and protein interaction networks. In particular, the availability of these datasets for many different diseases presents a ripe opportunity to use data-driven approaches to advance our current knowledge of disease relationships in a systematic way. As a matter of fact, very recently, Hu and Agarwal [10] presented an approach to determine disease relationships using only gene expression data. In order to obtain the disease correlations, the authors excluded genes which don't change meaningfully using an arbitrary threshold. They also did not take advantage of the plethora of protein interaction data available for the human system. Protein networks represent the physical processes taking place inside a cell and are essential to acquire a complete understanding of any biological condition such as disease.

Therefore, just as sequencing of genomes has enabled the reorganization of many species and provided quantitative metrics to appreciate their relationships, we believe an integrated approach combining both mRNA expression and protein interaction data will provide us a quantitative way to assess the correlation between diseases. Here, we present the first such systematic and integrated approach to explore the architecture of human diseases. In particular, we identified 4,620 functional modules analogous to important complexes and pathways in the human protein network and recorded how they varied in each of the 54 diseases using the mRNA expression data. This process provided a quantitative measure to describe the overall response of the human system to a given disease. Subsequently, we used these measures to identify 138 significant associations between diseases. We also discovered functional modules that are common to at least half of the diseases representing a common “disease-state” signature. These common disease-state modules were not only significantly enriched for genes that were known drugs targets, but their corresponding drugs were known to treat significantly more diseases than expected by chance highlighting their importance as therapeutic opportunities.

## Results/Discussion

**Figure 1:**
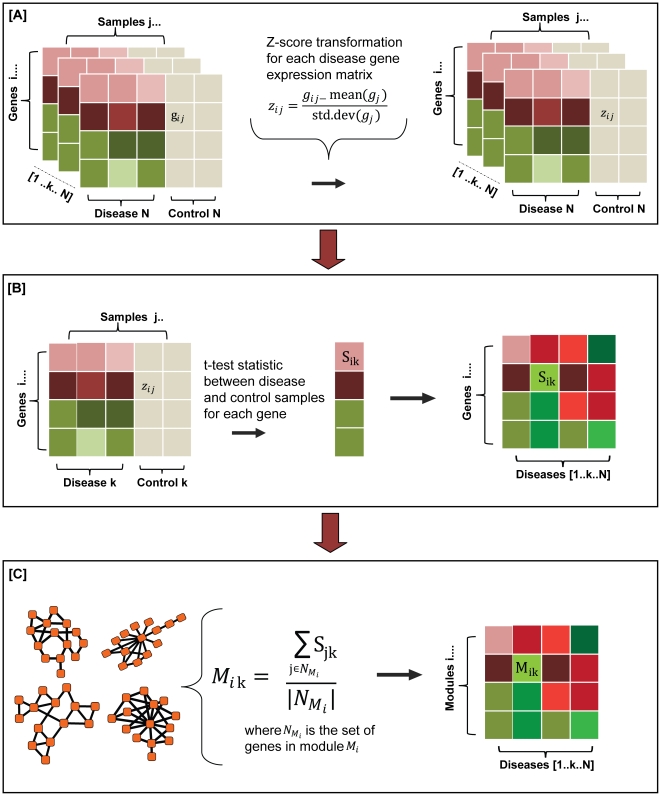
Overview of the process to generate the module response scores for each disease. (A) Normalization of the gene expression matrices through a Z-score transformation. In the gene expression matrix for a given disease k, g_ij_ represents the expression value of gene i in sample j, g_j_ corresponds to the whole set of gene expression values for a given sample j (j^th^ column) and z_ij_ corresponds to the z-score transformed gene expression value of gene i sample j. (B) Response score of a gene in a given disease. The response score of gene i in a disease k is the t-test statistic between the disease and control sample values for that gene. This score is represented as S_ik_. (C) Module response score calculation. The Module Response Score (MRS) of a given module i in a given disease k (M_ik_) is average of the response scores of its component genes. Detailed description of this process is provided in the Methods section.

Protein complexes and pathways are accountable for most processes in the cell. Accordingly, we can gauge the response of a cell system to a certain perturbation (such as disease) by the measuring changes in the expression levels of various functional modules of the system. To this end, we first generated a catalog of 4,620 functional modules by querying the large-scale human protein interaction network (see Methods). We then collected the mRNA expression arrays associated with each disease from the Gene Expression Omnibus (GEO) [11]. After several rounds of filtering the gene expression data for accuracy, reliability, and experimental context, we had microarrays representing 54 human diseases (see Methods and Table S1). Next, we combined the gene expression data and the 4,620 functional modules to generate a Module Response Score (MRS) for each module in each disease-state representing its activity level (see Methods). Specifically, positive MRS values correspond to modules that are up-regulated and negative MRS values identify modules that are down-regulated in the disease-state as compared to the control (healthy). Figure 1 gives an overview of the process to compute the MRS values for a given disease. In the end, we generated a matrix containing the MRS values for each module in each of the 54 diseases considered in this study. The relationships between different diseases were then ascertained by the Partial Spearman correlation coefficient of their MRS values (see Methods and Figure S1). Specifically, we calculated the Spearman correlation between two diseases conditioned on the responses of the functional modules in their respective control samples. The use of the Partial Spearman correlation coefficient instead of the generic Spearman correlation coefficient not only provided a quantitative metric to assess disease similarity but also explicitly factored out the possible dependencies between different gene-expression experiments due to their underlying tissue or cell types.

**Figure 2:**
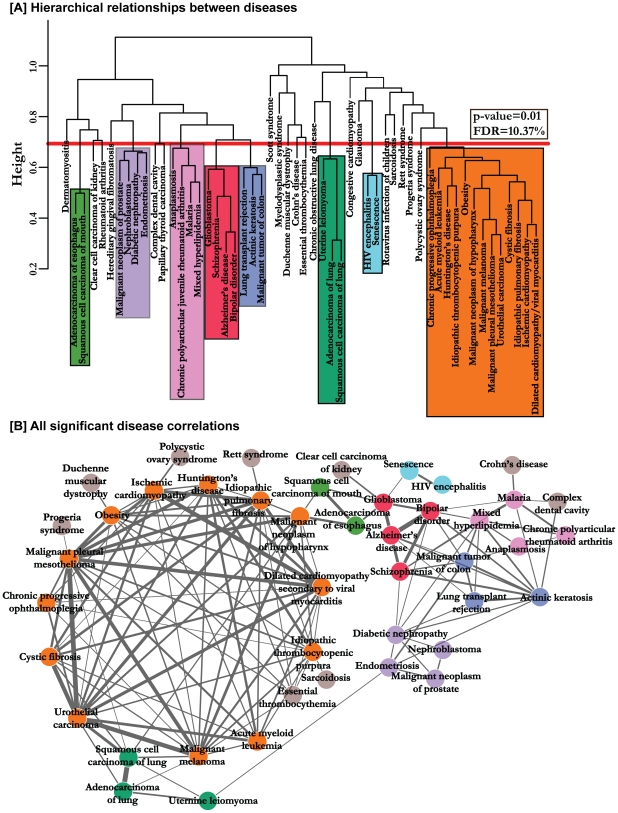
Significant disease-disease similarities. (A) Hierarchical clustering of the disease correlations. The distance between two diseases was defined to be (1-correlation coefficient) of the two diseases. The tree was constructed using the average method of hierarchical clustering. The red line corresponds to a *p*-value of 0.01 and FDR of 10.37% and, disease correlations below this line are considered significant. The different colors represent the various categories of significant disease correlations. (B) The network of all the 138 significant disease correlations. The colors correspond to significant disease correlation categories in (A). The nodes colored in grey are not marked in (A).

Figure 2(A) is the hierarchical clustering of diseases based on the correlations generated above. To assign significance to these associations, we randomized the gene to module assignments as well as the control and disease labels 100 times to generate a background distribution of disease correlations (see Methods). We then selected only those disease correlations that passed the *p*-value threshold of 0.01 (FDR = 10.37%) resulting in 138 significant disease-disease similarity relationships. Immediately, we see that many expected disease associations such as the brain disorders like Alzheimer's disease, Bipolar disorder and Schizophrenia are pooled together in one sub-branch. We also see many novel and hitherto unknown significant correlations such as the similarity between uterine leiomyoma and lung cancer. We also created a network representation to display all the 138 significant disease correlations (Figure 2(B)). In this network, the nodes are diseases, while the thickness of the edges between two diseases represents their strength of correlation. This abstraction allows us to pick additional significant disease associations that were missing in the hierarchical clustering. For example, Crohn's disease and Malaria share a significant disease correlation. A listing of all the significant disease correlations is provided in Table S2.

Although the 54 diseases considered in this study cover many categories of diseases ranging from cancers to cardiomyopathies, some categories of diseases such as cancer are over-represented as opposed to others such as infectious diseases. Ideally, we would like to explicitly correct for this bias by down-weighing over-represented classes. However, the principle behind organizing diseases into categories such as cancers, infectious diseases and others is not the same. For instance, diseases are classified as cancers if their underlying pathology consists of a group of cells that show uncontrolled growth, invasion of nearby cells and metastasis. On the other hand, infectious diseases relates to diseases which are caused by pathogens and have the potential to spread from person to person. Lack of a common organization scheme prevents us from explicitly correcting for the observed over-representation. Moreover, there is considerable heterogeneity even among diseases of the same category. For instance, the category of cancers covers a wide variety of diseases affecting many different cell types and having many different biological causes ranging from mutations caused by chemical carcinogens to bacterial and viral infection. This heterogeneity is seen even at the transcriptional level [12]. We also have observed this heterogeneity in the results of our study as all the 17 cancers considered in our analysis did not cluster together (Figure 2(A)). By combining both mRNA expression and protein interaction data, we are providing one of the first ways to compare and classify diseases systematically. The common organizing principle here is the molecular pathology of a given disease.

At the outset, we explored the genetic basis of the diseases in our study to explain and validate the observed disease correlations. Specifically, we aimed to test the hypothesis that diseases which are significantly associated through the MRS-based correlation coefficient also significantly shared disease genes. For this purpose, we collected a list of genes known to be associated with diseases, hereinafter as the Disease Gene List (see Methods). We found known gene variants associated with only 31 of the 54 diseases in our study resulting in an overall total of 465 possible pair-wise disease comparisons. A pair of diseases was considered to significantly share disease genes only if the Hypergeometric *p*-value of the overlap was less than 0.01. Eighty-two of the overall 465 comparisons significantly shared disease genes. On the other hand, only 73 of the 465 disease pairs were significantly associated using the MRS-based correlation coefficient. This gives rise to a contingency table as shown in Table 1 with a one-sided Fisher's Exact Test *p*-value of 0.033. It suggests that the genetic similarity between diseases significantly contributes to the molecular pathological disease similarity observed in this study. Lack of a strong *p*-value might be explained by the fact that the number of known disease genes are much higher for well-studied diseases like Schizophrenia (345 genes) as opposed to less well-studied diseases like Mixed hyperlipidemia (4 genes). Mapping of genes to diseases was also hindered due to fact that we used a very strict vocabulary to define diseases (see Methods). Finally, this result might also allude to the role of environment in disease causation and similarity. A few of the significant disease correlations which also significantly shared disease genes is provided in Table 2 and the complete list is provided in Table S3.

**Table 1:** Contingency table to evaluate the hypothesis that significant disease correlations also significantly shared disease genes.

Number of disease pairs		MRS-based disease correlations		
		Significant	Not significant	Totals
**Shared disease genes**	Significant	19	63	82
	Not significant	54	329	383
	**Totals**	73	392	465

We performed a one-sided Fisher's Exact Test on this table giving a *p*-value of 0.033.

**Table 2:** Genetic similarity between significant disease correlations.

Disease 1	Disease 2	Correlation	# shared disease genes	Disease 1 genes	Disease 2 genes	Hypergeometric *p*-value
Bipolar disorder	Schizophrenia	0.524	98	145	345	1.6E-23
Endometriosis	Malignant neoplasm of prostate	0.356	28	56	173	6.69E-11
Crohn's disease	Malaria	0.381	23	120	63	2.86E-9

This table shows only 3 of the 19 disease correlations significantly sharing at least one disease gene. The complete list is in Table S3. The “correlation” column indicates the calculated MRS-based correlation between the respective diseases. The number of genes whose variants are associated with any given disease was obtained from the Disease Gene List. We calculated the hypergeometric p-value by estimating the probability of seeing the observed overlap or more by chance accounting for the total number of disease genes present in our dataset (N = 2,104).

**Figure 3:**
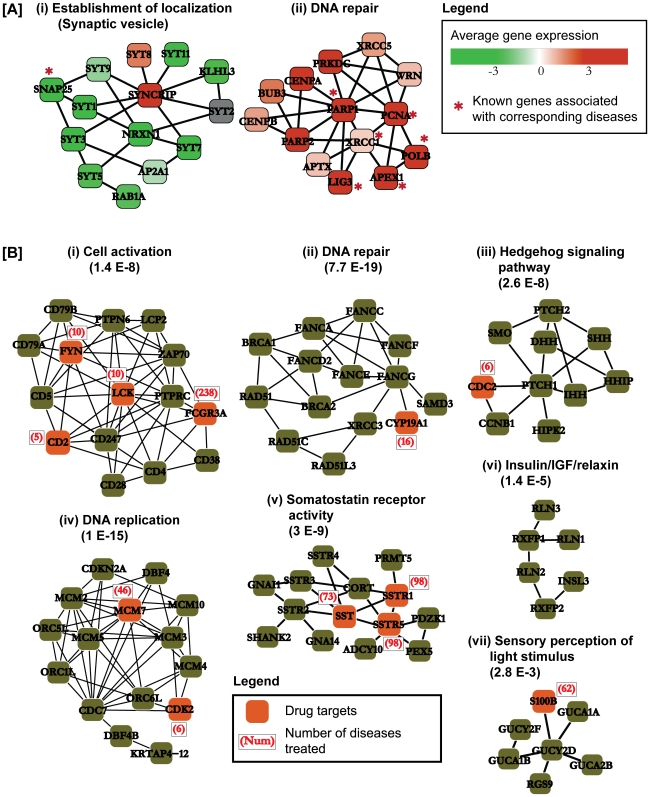
Underlying functional modules. (A) Two representative samples of functional modules. (i) The synaptic vesicle module is one of the most down-regulated modules among set of brain disorders: Alzheimer's disease, Bipolar disorder, Schizophrenia and Glioblastoma. (ii) The DNA repair module is one of the most up-regulated modules among the lung cancers and Uterine leioyomyoma. The colors of the nodes represent their average gene expression in their corresponding diseases. The genes marked with a red star next to them are genes with known variants associated with disease (see methods). (B) A representative sample of common disease “signature” modules. The genes colored in orange correspond to known drug targets. The functions for the modules were obtained by the functional enrichment tool of the DAVID database [45].

In order to further understand the biology behind the observed disease correlations, we examined some of their underlying functional modules. First, we analyzed the sub-branch of brain disorders, Alzheimer's disease (ALZ), Bipolar disorder (BIP), Schizophrenia (SCHZ), and Glioblastoma (GLIO), in the hierarchical representation of the disease correlations (Figure 2(A)) in more detail. Figure 3(A.i) corresponds to the synaptic vesicle and was one of most down-regulated modules in all four diseases (second lowest average MRS value). This module is a secretory organelle that stores neurotransmitters and releases them into the synapse. Loss of synaptic functions and more specifically, decreased expression of synaptic vesicle proteins such as SNAP-25 is one of the main effects of ALZ [13],[14]. Decreased synaptic function has also been observed for both BIP and SCHZ [15],[16]. In particular, the levels of protein SNAP-25 was shown to be reduced in both BIP and SCHZ [17]. The function of this module in GLIO is still to be explored. Uterine leiomyomas (UTL) are benign tumors affecting the uterus. As shown in Figure 2(A), UTL shares a strong correlation with lung cancers. Figure 3(A.ii) corresponds to the DNA repair pathway which had the highest average MRS value for the three diseases. Polymorphisms in the genes involved in the DNA repair pathway such as PCNA, POLB have been associated with increased risk of lung cancer [18]. Moreover, the Arg399Glu allele of the XRCC1 gene has been shown to be a risk factor for lung adenocarcinoma [19] and lung squamous cell carcinoma [20]. Surprisingly, the same Arg399Glu polymorphism in the XRCC1 gene has also been associated with an increased risk of UTLs [21] giving causal genetic evidence for the correlation we observed between the diseases using microarray-based molecular pathological measurements.

Knowledge of a comprehensive disease-similarity tree (network) based on molecular data could possibly be used in finding new uses for existing drugs. Similar diseases share similar molecular phenotypes and could potentially be treated by similar drugs. To explore this avenue, we collected a list of drugs, their corresponding target genes and the diseases they are known to treat (US FDA approved indications) or off-label uses. This information was obtained from the RxNorm from National Library of Medicine [22], DrugBank [23], National Drug File Reference Terminology (ND-FRT) [24] and MicroMedex [25]. Overall, 17 of the 138 significant disease correlations shared at least one drug in common and 14 of them had a significant Hypergeometric *p*-value less than 0.01 (Table 3, Table S4). For instance, we found that the FDA approved drug Flouroucil, used to treat Actinic keratosis, has been shown to have positive indications for treating Malignant tumor of the colon [25]. Similarly, the drug Doxorubicin is FDA approved to treat both Urothelial carcinoma and Acute myeloid leukemia [25]. This number is a conservative estimate as the list of drugs used here is incomplete. Moreover, we used a very specific vocabulary to define diseases (see Methods) and accordingly mapped drugs to them. For instance, we found many drugs treating lung cancer; however in many cases, our combined knowledge base doesn't specify whether the cancer was an adenocarcinoma or a squamous cell carcinoma. In those cases, we excluded the drug from our consideration. A caveat to this approach is that drugs can be shared between diseases mainly because the corresponding diseases belong to the same category. For instance, drugs can be shared between two cancers etc. As a result, it is difficult to differentiate whether two diseases shared drugs due to the similarity in their molecular pathology or due to their underlying disease type. Moreover, the chemical similarity between drugs can also affect the reported *p*-values.

**Table 3:** Shared drugs among significant disease correlations.

Disease 1	Disease 2	Correlation	# shared Drugs	Drugs 1	Drugs 2	Hypergeometric *p*-value
Actinic keratosis	Malignant tumor of colon	0.428	1	9	13	4.40E-04
Bipolar disorder	Schizophrenia	0.524	16	37	106	3.35E-17
Acute myeloid leukemia	Urothelial carcinoma	0.496	2	31	10	7.00E-05

This table shows only 3 of the 14 significant disease correlations that significantly shared at least one drug. The complete list is in Table S4. The “correlation” column indicates the calculated correlation between the respective diseases. We calculated the hypergeometric p-value by estimating probability of seeing the observed overlap or more by chance accounting for the total number of drugs in our knowledge base (N = 3,536).

Another consequence of elucidating and quantifying the response of the cell system to a disease is that we can use this methodology to find modules that are generally dysregulated (activated or repressed) in the disease-state. In other words, we used the MRS values to characterize a common “signature” across disease-states. In order to generate the set of modules that are commonly dysregulated in the 54 diseases considered in this study, we used a two-fold approach. Firstly, a module was selected if the median of its absolute MRS values across all diseases was significantly higher than expected at random. We generated a random background distribution of median scores by shuffling the gene to module assignments (see Methods). Overall, at a *p*-value of 0.01 and associated FDR of 16.15%, we selected 286 modules. We then filtered the above set of 286 modules to only include those modules which were significantly differentially expressed in many diseases. A module was determined to be significantly differentially expressed in a given disease if the absolute value of its MRS was above 1.5 (*p*-value = 0.028). Finally, we selected 59 modules that were significantly differentially expressed in 20 or more diseases as the common disease state signature. These modules were not only dysregulated in at least half of the diseases each but were also significantly differentially expressed in more than 20 diseases. Moreover, these 59 modules taken together were dysregulated in 45 of the 54 diseases in our study. Figure S2 shows the combined illustration of all the 59 modules. They were mainly enriched for the functions of immune system response (*p*-value = 6E-70) and DNA repair (*p*-value = 4.1E-30). A representative sample of 7 modules is shown in Figure 3(B.iii–vii).

We investigated the 59 modules further by searching for known drug target genes/proteins. We obtained the list of drugs and their corresponding targets from the DrugBank database [23]. Overall, 70 genes/proteins within the 59 signature pathways were identified as targets of known drugs giving a Hypergeometric *p*-value of 1.8E-11. Thus, the set of the signature modules was significantly enriched for drug target genes compared to that expected by chance. We then predicted that other genes/proteins in these modules would also serve as prime candidates for designing new drugs. Most existing drug target genes usually fall into a comparatively small set of gene families such as G protein coupled receptors, serine proteases etc [26]. Hence, new drug targets can be found by exploring other members of the protein families of the existing drug targets. We explored the 59 signature modules for genes which belonged to the same protein families as known drug target genes. For that purpose, we obtained a list of genes and their corresponding families and sub-families from the PANTHER database [27]. Overall, we found 241 genes among a total of 450 genes in the signature modules sharing the same protein families as the known drug target genes compared to a total of only 3,520 such genes in the whole human PPI giving a Hypergeometric *p*-value of 1.47E-12. Therefore, the 59 signature modules were also significantly enriched for druggable genes. Further, we also counted the number of distinct diseases that are known to be treated by the drugs corresponding to each of the 70 known drug targets. We observed that drugs targeting these 70 genes are known to treat an average of 65 diseases each compared to an average of ∼42 diseases for all known drug targets (*p*-value = 0.02). These results provide evidence that the genes in the signature modules are more likely to be good drug targets and drugs that target these proteins are more likely to treat many diseases. Yildirim *et al.* [28] showed that most drugs seemed to be palliative and only cured the symptoms of the diseases rather than the diseases themselves. Therefore, the enrichment for drug target genes which treat many diseases might be due to the shared symptoms of the diseases.

In summary, this study demonstrates the value of an integrated approach in revealing disease relationships and the resultant opportunities for therapeutic applications. Looking forward, we aim to incorporate more gene expression data from GEO and other similar repositories, and expand the set of diseases in our disease-similarity network.

## Methods

### Gene expression dataset

The gene expression data used in this analysis was obtained from the NCBI Gene Expression Omnibus (GEO) [11]. In this study, we restricted to using only those microarrays that were curated and reported in the GEO Datasets (or GDS). We selected for microarrays that were assigned to human disease conditions. These assignments were made by the method explained in Butte *et al.* [29]. Briefly, the experimental context of a collection of microarrays from GEO (or GEO Series, GSE) can be obtained from the Medical Subject Headings (MeSH) [9] terms associated with the records of corresponding publications in PUBMED. Subsequently, the MeSH terms were connected to disease concepts using the Unified Medical Language System (UMLS) [30]. The GDS curation provided more details such as the tissue or biological substance from which the samples were derived. We only included those GSEs in which both disease as well as their corresponding control condition was measured in the same tissue (cell type) in the same experiment, using a previously described method [31]. We also manually selected for GSEs in which the control was a healthy sample. Further, we removed all GSEs that included time-series data to avoid complications arising due to temporal changes in gene expression. For consistency, we also restricted the GSEs to only those arrays which used Affymetrix Gene Chip Human Genome U133 Array Set HG-U133A or U95 Version 2 platforms, which are among the most commonly used platforms, mapping to current gene identifiers as previously described [32]. As both of these platforms have shared probe-sets, the bias of the platform used on the overall analysis would be reduced considerably. We subsequently selected GSEs that had at least two disease samples and two control samples. GEO contains some experiments (GSEs) that have gene expression measurements for more than one disease but share the same control measurements. Such measurements might induce correlations between their component diseases, which are not necessarily biological. Thus, to avoid bias, in all such cases, we included only one representative disease for each set of control samples in contrast to Hu *et al.* [10]. This entire process yielded 54 diseases for our final analysis.

### Protein interaction data

The protein-protein interaction (PPI) data for human was obtained from the Human Protein Reference Database (HPRD) [33]. This database contains PPI obtained from the two high-throughput yeast two-hybrid experiments [34],[35] as well as through literature curation. Further, HPRD contains the maximum number of PPI of any of publicly available literature-derived databases for human PPI [36]. We filtered the PPI for human proteins that had a corresponding Entrez Gene ID, yielding 34,998 unique protein interactions spanning 9303 proteins in human. Previously, Sharan *et al.* [37] presented the PathBLAST family of network alignment tools. Briefly, these methods help identify conserved modules between protein networks of two (or more) species. Suthram *et al.* [38] also used it effectively to identify dense subnetworks corresponding to functional modules within a protein network of a single species. Applying the same approach here, we identified 4,620 functional modules in the human PPI network.

### Module Response Score (MRS) generation

First, we normalized the gene expression data in each microarray sample (disease state or control) using the Z-score transformation. This transformation allows for the direct comparison of gene expression values across various microarray samples and diseases. Next, we computed the activity level of a gene i in disease k as the t-test statistic (S_ik_) of its Z-transformed score between the disease and the control samples for each disease. In cases where there was more than one experiment (or GEO Series) for a given disease, we employed a meta-analysis technique using linear regression to obtain a combined t-test statistic. This approach takes into account the variations between different experiments in the calculation of the gene activity score (S_ik_) (see section below). Finally, the module response score (M_ik_) for each module i in a disease k is assigned to be the mean of the gene activity scores (S_ik_) of its component genes. In the end, we obtained a vector of module response scores (M_ik_) for each disease.

### Combined t-test statistic for multiple experiments

The t-test statistic between two conditions can be represented using linear regression. For instance, let Y_i_ and X_i_ be gene expression values and disease state (disease has a value of 1 and control a value of 0), respectively. Then, we have a linear regression model as follows:
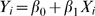
where 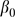 and 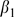 are the parameters of the model. The t-test statistic when estimating the value of 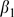 is the same as the standard t-test statistic between disease and control states. The advantage of the linear regression model is that we can add more terms to the model to account for other sources of variation such as the experiment number. In the present work, we expanded on the above model as follows:
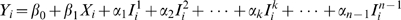
where *n* is the number of different experiments for a given disease and, 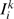 is an indicator variable which is 1 if the i'^th^ gene expression measurement is from the experiment number *k*. Again, the 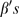 and 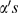 are the parameters of the model which need to be estimated. The addition of the new terms allows for explicitly accounting for the effect of the experiment on the gene expression value. This approach is similar to a mixed effects model for adjusting for the within-experiment dependencies, but is more aggressive in removing such effects. Consequently, the t-test statistic in the estimation of 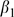 will now be a combined metric for the different studies.

### Partial correlation

The partial correlation coefficient gives the correlation between two variables, say *x* and *y*, keeping a third variable, *z*, constant. This method tries to measure the similarity between *x* and *y*, over and above that caused by their common dependency on *z*. The partial correlation can be calculated as follows:
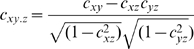



The above formula can be expanded to condition on two variables as follows:
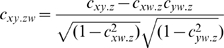
In this study, we calculated the Partial Spearman correlation between two diseases conditioned on the responses of the functional modules in their respective control samples. The response of a functional module in the control samples for a given disease was calculated as the mean of the z-transformed scores of its component genes. As a result, the Partial Spearman correlation coefficient provided a quantitative metric to assess disease similarity and also explicitly factored out the possible dependencies between different gene-expression experiments due to their underlying tissue or cell types [39]. Our approach is consistent with the findings by Dudley *et al.* [40] that the disease signal in the GEO datasets is stronger than the tissue signal and hence, implying that the observed disease correlations reflect true biology. We used the R script provided by Kim *et al.* [41] for calculating the Partial Spearman correlation between two diseases.

### Randomization procedure

To assign significance to the observed disease correlations, we created a background distribution of disease correlations expected at random. First, we randomized the gene to module assignments. We envisioned the gene to module assignments as a bi-partite graph (Figure S3) where there exists a link between a gene and a module if that gene is a member of that module. We then randomized the graph by randomly swapping links. This process preserved the number of modules, the number of genes assigned to a module as well as the number of modules a given gene belongs to. Next, we also shuffled the disease and the control sample labels. We then calculated the MRS values for the modules using the randomized data and computed the corresponding disease correlations. Finally, we repeated the whole process 100 times to create a background distribution of disease correlations.

### Disease gene list generation

We built a comprehensive disease-associated gene database, referred to as the Disease Gene List, by collecting genes known to be associated with various diseases from literature curation and large databases. In particular, we first curated 37,953 disease Single Nucleotide Polymorphism (SNP) associations from 2,679 papers, mapping 10,167 specific SNPs from the SNP Database (dbSNP) to 748 diseases and phenotypes. We then annotated each SNP with its corresponding gene(s) using dbSNP (Chen and Butte, unpublished data). Next, we extracted genes that are significantly associated with diseases in Genetic Association Database (GAD) [42]. These consisted of associations that were reported to be positive at least once. We also collected genes that are associated with disorders in the Online Mendelian Inheritance in Man (OMIM) [5]. Lastly, we retrieved genes that are associated with diseases in the professional version of Human Gene Mutation Database (HGMD) [43]. Finally, we combined disease genes obtained from the above four different sources by relating them to Entrez gene IDs and removing outdated Gene IDs using AILUN [32].

### Module visualization

The module figures in the paper were drawn using the Cytoscape software [44].

## Supporting Information

Figure S1 Schematic of the calculation of the Partial Spearman correlation between two diseases.
(0.49 MB EPS)

Figure S2 Combined representation of the 59 common disease-state “signature” modules. The genes marked in orange are known drug target genes.
(2.35 MB EPS)

Figure S3 Graphical representation of the genes to modules assignments.
(0.40 MB EPS)

Table S1 List of the 54 diseases considered in this study.
(0.02 MB XLS)

Table S2 List of the 138 significant disease correlations.
(0.03 MB XLS)

Table S3 Genetic similarity between significant disease correlations. This table shows the 19 disease correlations significantly sharing at least one disease gene. The number of genes whose variants are associated with any given disease was obtained from the Disease Gene List. We calculated the hypergeometric p-value by estimating the probability of seeing the observed overlap or more by chance accounting for the total number of disease genes present in our dataset (N = 2,104).
(0.02 MB XLS)

Table S4 This table shows the 14 significant disease correlations that significantly shared at least one drug. We calculated the hypergeometric p-value by estimating probability of seeing the observed overlap or more by chance accounting for the total number of drugs in our knowledge base (N = 3,536).
(0.02 MB XLS)

## References

[1] Kalaria R (2002). Similarities between Alzheimer's disease and vascular dementia.. J Neurol Sci.

[2] Lage K, Karlberg EO, Storling ZM, Olason PI, Pedersen AG (2007). A human phenome-interactome network of protein complexes implicated in genetic disorders.. Nat Biotechnol.

[3] Wu X, Liu Q, Jiang R (2009). Align human interactome with phenome to identify causative genes and networks underlying disease families.. Bioinformatics.

[4] Tao Y, Sam L, Li J, Friedman C, Lussier YA (2007). Information theory applied to the sparse gene ontology annotation network to predict novel gene function.. Bioinformatics.

[5] Amberger J, Bocchini CA, Scott AF, Hamosh A (2009). McKusick's Online Mendelian Inheritance in Man (OMIM).. Nucleic Acids Res.

[6] Goh KI, Cusick ME, Valle D, Childs B, Vidal M (2007). The human disease network.. Proc Natl Acad Sci U S A.

[7] van Driel MA, Bruggeman J, Vriend G, Brunner HG, Leunissen JA (2006). A text-mining analysis of the human phenome.. Eur J Hum Genet.

[8] Liu YI, Wise PH, Butte AJ (2009). The “etiome”: identification and clustering of human disease etiological factors.. BMC Bioinformatics.

[9] Fowler J, Kouramajian V, Maram S, Devadhar V (1995). Automated MeSH indexing of the World-Wide Web.. Proc Annu Symp Comput Appl Med Care.

[10] Hu G, Agarwal P (2009). Human disease-drug network based on genomic expression profiles.. PLoS ONE.

[11] Barrett T, Troup DB, Wilhite SE, Ledoux P, Rudnev D (2007). NCBI GEO: mining tens of millions of expression profiles–database and tools update.. Nucleic Acids Res.

[12] Rhodes DR, Yu J, Shanker K, Deshpande N, Varambally R (2004). Large-scale meta-analysis of cancer microarray data identifies common transcriptional profiles of neoplastic transformation and progression.. Proc Natl Acad Sci U S A.

[13] Coleman PD, Yao PJ (2003). Synaptic slaughter in Alzheimer's disease.. Neurobiol Aging.

[14] Shimohama S, Kamiya S, Taniguchi T, Akagawa K, Kimura J (1997). Differential involvement of synaptic vesicle and presynaptic plasma membrane proteins in Alzheimer's disease.. Biochem Biophys Res Commun.

[15] Vawter MP, Thatcher L, Usen N, Hyde TM, Kleinman JE (2002). Reduction of synapsin in the hippocampus of patients with bipolar disorder and schizophrenia.. Mol Psychiatry.

[16] Sokolov BP, Tcherepanov AA, Haroutunian V, Davis KL (2000). Levels of mRNAs encoding synaptic vesicle and synaptic plasma membrane proteins in the temporal cortex of elderly schizophrenic patients.. Biol Psychiatry.

[17] Scherk H, Backens M, Zill P, Schneider-Axmann T, Wobrock T (2008). SNAP-25 genotype influences NAA/Cho in left hippocampus.. J Neural Transm.

[18] Zienolddiny S, Campa D, Lind H, Ryberg D, Skaug V (2006). Polymorphisms of DNA repair genes and risk of non-small cell lung cancer.. Carcinogenesis.

[19] Divine KK, Gilliland FD, Crowell RE, Stidley CA, Bocklage TJ (2001). The XRCC1 399 glutamine allele is a risk factor for adenocarcinoma of the lung.. Mutat Res.

[20] Park JY, Lee SY, Jeon HS, Bae NC, Chae SC (2002). Polymorphism of the DNA repair gene XRCC1 and risk of primary lung cancer.. Cancer Epidemiol Biomarkers Prev.

[21] Jeon YT, Kim JW, Park NH, Song YS, Kang SB (2005). DNA repair gene XRCC1 Arg399Gln polymorphism is associated with increased risk of uterine leiomyoma.. Hum Reprod.

[22] Parrish F, Do N, Bouhaddou O, Warnekar P (2006). Implementation of RxNorm as a terminology mediation standard for exchanging pharmacy medication between federal agencies.. AMIA Annu Symp Proc.

[23] Wishart DS, Knox C, Guo AC, Cheng D, Shrivastava S (2008). DrugBank: a knowledgebase for drugs, drug actions and drug targets.. Nucleic Acids Res.

[24] Brown SH, Elkin PL, Rosenbloom ST, Husser C, Bauer BA (2004). VA National Drug File Reference Terminology: a cross-institutional content coverage study.. Stud Health Technol Inform.

[25] DrugDex (2007). DRUGDEX System..

[26] Hopkins AL, Groom CR (2002). The druggable genome.. Nat Rev Drug Discov.

[27] Mi H, Guo N, Kejariwal A, Thomas PD (2007). PANTHER version 6: protein sequence and function evolution data with expanded representation of biological pathways.. Nucleic Acids Res.

[28] Yildirim MA, Goh KI, Cusick ME, Barabasi AL, Vidal M (2007). Drug-target network.. Nat Biotechnol.

[29] Butte AJ, Chen R (2006). Finding disease-related genomic experiments within an international repository: first steps in translational bioinformatics.. Annual Symposium of the American Medical Informatics Association Proceedings.

[30] Bodenreider O (2004). The Unified Medical Language System (UMLS): integrating biomedical terminology.. Nucleic Acids Res.

[31] Dudley J, Butte AJ (2008). Enabling integrative genomic analysis of high-impact human diseases through text mining.. Pac Symp Biocomput.

[32] Chen R, Li L, Butte AJ (2007). AILUN: reannotating gene expression data automatically.. Nat Methods.

[33] Keshava Prasad TS, Goel R, Kandasamy K, Keerthikumar S, Kumar S (2009). Human Protein Reference Database–2009 update.. Nucleic Acids Res.

[34] Rual JF, Venkatesan K, Hao T, Hirozane-Kishikawa T, Dricot A (2005). Towards a proteome-scale map of the human protein-protein interaction network.. Nature.

[35] Stelzl U, Worm U, Lalowski M, Haenig C, Brembeck FH (2005). A human protein-protein interaction network: a resource for annotating the proteome.. Cell.

[36] Mathivanan S, Periaswamy B, Gandhi TK, Kandasamy K, Suresh S (2006). An evaluation of human protein-protein interaction data in the public domain.. BMC Bioinformatics.

[37] Sharan R, Suthram S, Kelley RM, Kuhn T, McCuine S (2005). Conserved patterns of protein interaction in multiple species.. Proc Natl Acad Sci U S A.

[38] Suthram S, Sittler T, Ideker T (2005). The Plasmodium protein network diverges from those of other eukaryotes.. Nature.

[39] Suthram S, Shlomi T, Ruppin E, Sharan R, Ideker T (2006). A direct comparison of protein interaction confidence assignment schemes.. BMC Bioinformatics.

[40] Dudley JT, Tibshirani R, Deshpande T, Butte AJ (2009). Disease signatures are robust across tissues and experiments.. Mol Syst Biol.

[41] Kim SH, Yi SV (2007). Understanding relationship between sequence and functional evolution in yeast proteins.. Genetica.

[42] Becker KG, Barnes KC, Bright TJ, Wang SA (2004). The genetic association database.. Nat Genet.

[43] Stenson PD, Mort M, Ball EV, Howells K, Phillips AD (2009). The Human Gene Mutation Database: 2008 update.. Genome Med.

[44] Shannon P, Markiel A, Ozier O, Baliga NS, Wang JT (2003). Cytoscape: a software environment for integrated models of biomolecular interaction networks.. Genome Res.

[45] Huang da W, Sherman BT, Lempicki RA (2009). Systematic and integrative analysis of large gene lists using DAVID bioinformatics resources.. Nat Protoc.

